# Comprehensive Genomic Profiling of Cutaneous Adnexal Carcinomas: A Genomic Landscape Study

**DOI:** 10.3390/dermatopathology13020015

**Published:** 2026-03-30

**Authors:** Maroun Bou Zerdan, Kevin T. Jamouss, Alexandre Maalouf, Rita Moukarzel, Tanishq Chhabra, Daniel J. Zaccarini, Dean Pavlick, Natalie Danziger, Jeffrey Ross

**Affiliations:** 1 Department of Internal Medicine, SUNY Upstate Medical University, Syracuse, NY 13210, USA; 2 Department of Hematology and Oncology, Winship Cancer Institute, Atlanta, GA 30322, USA; 3 Department of Internal Medicine, University of Massachusetts Chan Medical School—Baystate, Springfield, MA 01199, USA; 4 Department of Pediatrics, Children’s National Hospital, Washington, DC 20010, USA; 5 Department of Pathology, Montefiore Medical Center, Bronx, NY 10467, USA; 6 Department of Medicine, Hamdard Institute of Medical Sciences and Research, New Delhi 110062, India; 7 Department of Pathology, SUNY Upstate Medical University, Syracuse, NY 13210, USA; 8 Foundation Medicine, Inc., Morrisville, NC 27560, USA

**Keywords:** adnexal carcinoma, sweat glands, dermatopathology, genomic profiling

## Abstract

Cutaneous adnexal carcinomas (CACs) comprise a diverse group of malignant tumors that show morphological differentiation toward one of the four main adnexal structures in normal skin: hair follicles, sebaceous glands, sweat-apocrine glands, and sweat-eccrine glands. These tumors can arise sporadically or may be associated with rare genetic syndromes. A total of 276 CACs cases underwent hybrid capture-based comprehensive genomic profiling (CGP) to assess all classes of genomic alterations (GA). Sequencing data were used to determine microsatellite instability (MSI) status, tumor mutational burden (TMB), genomic loss of heterozygosity (gLOH), genomic ancestry, and COSMIC mutational signatures. PD-L1 expression was evaluated by immunohistochemistry (TPS; Dako 22C3). Statistical analyses were performed using Fisher’s exact test, with false discovery rate correction via the Benjamini–Hochberg method. Sequencing was performed on primary cutaneous tumors in 131 cases (47.4%) and on local recurrence or metastatic site biopsies in 145 cases (52.5%). Across all groups, there was a male predominance (64–81%) and similar mean ages (59–63 years), with apocrine (APO) tumors occurring in older patients than eccrine (ECC) tumors (72 vs. 62 years; *p* = 0.001). Histologically, 173 tumors (62.7%) were sweat gland-derived (SWT), 55 (19.9%) sebaceous gland-derived (SEB), 14 (5.1%) hair follicle-derived (HRF), and 34 (12.3%) unclassified (UNK). Among SWT tumors, 150 (86.7%) were eccrine and 23 (13.3%) apocrine. SWT tumors included digital papillary adenocarcinomas (DPA, 6.9%), mucinous carcinomas (MC, 6.3%), porocarcinomas (POR, 11.0%), spiradenocarcinomas (SPR, 8.1%), syringoadenocarcinomas (SRNG, 5.8%), and 77 (44.5%) unclassified cases. The number of GA per tumor was highest in SEB compared with SWT tumors (7.9 vs. 4.9; *p* = 0.005) and lowest in DPA (2.1 vs. 5.0 in non-DPA; *p* = 0.03). No differences in ancestry distribution were observed. Compared with SWT tumors, SEB tumors exhibited higher frequencies of RB1 (38.2% vs. 8.1%; *p* < 0.0001) and TP53 alterations (76.4% vs. 43.4%; *p* = 0.0002), suggesting potential neuroendocrine differentiation. MC tumors showed significantly higher PTCH1 alterations than non-MC tumors (36.4% vs. 1.8%; *p* = 0.044). MSI-high status was most frequent in SEB tumors compared with all other groups (15.7% vs. 1.2%; *p* = 0.005), and gLOH > 16% was also more common in SEB than SWT tumors (19.6% vs. 7.2%; *p* = 0.081). The MMR signature occurred more frequently in SEB than SWT tumors (32.0% vs. 2.1%; *p* = 0.005). Mean TMB was elevated across most CACs types, ranging from 10.4 mutations/Mb in HRF to 38.8 mutations/Mb in MC, with the exceptions of APO (2.7 mut/Mb; *p* = 0.001) and DPA (1.4 mut/Mb; *p* = 0.003). PD-L1 expression was generally low and did not differ significantly between SWT and SEB tumors (37.0% vs. 33.3%; NS). Given the limited data on CAC treatment, this study provides a catalog of commonly observed GA. SEB tumors exhibited the highest frequency of genomic alterations. Prospective clinical trials are needed to determine the prognostic and predictive value of CAC-specific biomarkers for immune checkpoint inhibitor (ICI) response, which is essential for integrating novel therapies into the evolving treatment landscape.

## Simple Summary

Cutaneous adnexal carcinomas are rare skin cancers that arise from structures such as hair follicles, oil glands, and sweat glands. These tumors can look very different under the microscope, and some may signal inherited conditions, making accurate diagnosis important. In this study, we analyzed the genetic makeup of 276 such cancers to better understand how they behave and how they might respond to new treatments. We found that tumors from oil glands carried more genetic changes, showed signs of high instability, and often involved genes linked to neuroendocrine features. Certain sweat gland tumors had unique gene alterations, while others had very few changes overall. Most tumors had a high number of mutations, suggesting they may respond to immunotherapy. These findings give new insight into the biology of these uncommon cancers and may help guide future research and patient care.

## 1. Introduction

Cutaneous adnexal tumors (CATs) are a varied group of neoplasms that originate from or differentiate towards the adnexal structures of the skin, including hair follicles, sebaceous glands, and sweat glands (both eccrine and apocrine). Cutaneous adnexal carcinomas (CACs) are rare and can be challenging to diagnose due to their morphological diversity and overlap with other skin neoplasms [1,2]. The classification of CACs is complex, with entities often exhibiting overlapping histological features and perplexing pathologists. This overlap can complicate accurate diagnosis, necessitating a combination of clinical, histopathological, and immunohistochemical evaluations [3,4]. We have used comprehensive genomic profiling (CGP) in evaluating these tumors. CGP can paint us a picture of all classes of genomic alterations, including microsatellite instability (MSI), tumor mutational burden (TMB), and specific gene fusions, which allows for a more precise molecular characterization of CACs, facilitating the prognostication of these tumors, identification of potential therapeutic targets and development of personalized treatment strategies [1,5]. Understanding the genomic alterations in CACs is crucial for therapeutic targeting. Recent studies have identified various oncogenic drivers and tumor suppressor alterations, such as mutations in *TP53*, *RB1*, and *PTCH1*, which can inform treatment strategies. For example, sebaceous gland-derived CACs have been shown to have higher frequencies of *RB1* and *TP53* genomic alterations, whose co-occurrence suggests potential neuroendocrine differentiation [6,7]. While the co-occurrence of these mutations is characteristic of neuroendocrine carcinomas in other sites, this association is still not specific in sebaceous tumors [8]. Our study corroborates the fact that SEB CACs have higher RB1 and TP53 mutations, thus making these genomic alterations possible therapeutic targets. The tumor mutational burden (TMB) is also notably high in sebaceous gland-derived CACs. Thus, suggesting a potential for immunotherapy as high TMB has been associated with better responses to immune checkpoint inhibitors, making it a promising therapeutic target [7]. Given the absence of prospective clinical outcome data within this dataset, this study is intended to provide a descriptive and hypothesis-generating overview of the genomic landscape of advanced CACs.

## 2. Materials and Methods

Approval for this study, including a waiver of informed consent and a Health Insurance Portability and Accountability Act (HIPAA) waiver of authorization, was obtained from the Western Institutional Review Board (Protocol No. 20152817), ensuring that all procedures complied with ethical standards for human research. A total of 276 cases of metastatic or CACs were selected from the Foundation Medicine (Cambridge, MA, USA) data biobank for comprehensive molecular analysis. Because the dataset included both primary and metastatic specimens submitted for clinical profiling, analyses were not stratified by site of biopsy. Therefore, comparisons across histologic subtypes reflect aggregate genomic findings and may be influenced by differences in disease stage or sampling context.

Tumor DNA was isolated from formalin-fixed, paraffin-embedded (FFPE) tissue specimens, which were either primary tumors or metastatic/recurrent lesions. FFPE tissue was first macrodissected to enrich for tumor content, followed by extraction of genomic DNA using standardized protocols optimized for recovery from formalin-fixed samples. Extracted DNA then underwent library preparation, during which DNA fragments were end-repaired, A-tailed, and ligated to sequencing adapters containing unique molecular identifiers (UMIs) to enable error correction and accurate detection of low-frequency variants. Targeted hybrid capture was performed to enrich for cancer-related genes included in the assay’s panel, ensuring high coverage of clinically and biologically relevant genomic loci.

Next-generation sequencing (NGS) was carried out using a validated platform with uniform, high-depth coverage (median 250×), which provides sufficient sensitivity to detect somatic alterations present at low allelic fractions. Following sequencing, bioinformatic processing was conducted using a clinically validated pipeline. Base substitutions (single-nucleotide variants), small insertions and deletions (indels), copy-number alterations, and gene rearrangements were identified, with stringent quality control metrics applied at each step to ensure reliable variant calls. Variants were required to meet predefined thresholds for minimum depth of coverage, base quality score, mapping quality, and allele frequency. Germline variants were filtered out using matched normal controls, where available, and population variant databases, ensuring the reported alterations reflected true somatic events.

Tumor mutational burden (TMB) was calculated as the number of somatic, coding, base substitution and indel mutations per megabase of sequenced coding genome, providing a quantitative measure of overall mutational load. Microsatellite instability (MSI) was assessed by examining variability in length across hundreds of microsatellite loci, allowing classification of tumors as MSI-high or MSI-stable. Genomic loss of heterozygosity (gLOH) was computed to assess the fraction of the genome exhibiting allelic imbalance, which can indicate homologous recombination deficiency. COSMIC mutational signatures were extracted from the mutational profiles to infer underlying mutagenic processes. Additionally, genomic ancestry was predicted based on variant allele frequencies at population-informative loci. PD-L1 expression was measured using immunohistochemistry (IHC) with the Dako 22C3 assay and reported as the tumor proportion score (TPS) [9,10,11,12,13,14].

All genomic alterations were interpreted according to pre-established, clinically validated thresholds. This included ensuring sufficient sequencing depth and quality for each variant, as well as confirming that allelic fractions met the assay’s sensitivity specifications. Only high-confidence somatic alterations were included in downstream analyses.

For statistical evaluation, Fisher’s exact test was employed to compare the frequency of genomic alterations across tumor subgroups, providing robust analysis for categorical data even with small sample sizes. To account for multiple hypothesis testing and control the false discovery rate, the Benjamini–Hochberg method was applied, thereby minimizing the likelihood of type I errors. This rigorous analytical framework ensured that all observed associations between tumor type and genomic features were statistically reliable and biologically meaningful.

## 3. Results

A total of 276 cases of CACs underwent comprehensive genomic profiling (CGP), including analysis of genomic alterations (GA), microsatellite instability (MSI) status, tumor mutational burden (TMB), genomic loss of heterozygosity (gLOH), and PD-L1 expression. Of these, 131 cases (47.4%) were sequenced from the primary cutaneous tumor and 145 (52.5%) from local recurrence or metastatic biopsies. The cohort demonstrated a male predominance (64–81%) with a mean age range of 59–63 years; notably, apocrine tumors (APO) were significantly older than eccrine tumors (ECC) (72 vs. 62 years; *p* = 0.001).

Of the cases, 173 (62.7%) were sweat gland (SWT)-derived, 55 (19.9%) sebaceous gland (SEB)-derived, 14 (5.1%) hair follicle (HRF)-derived, and 34 (12.3%) unclassified. Among SWT tumors, 150 (86.7%) were ECC and 23 (13.3%) APO. Further subclassification of SWT tumors identified 12 digital papillary adenocarcinomas (DPA), 11 mucinous carcinomas (MC), 19 porocarcinomas (POR), 14 spiradenocarcinomas (SPR), 10 syringoadenocarcinomas (SRNG), and 77 unclassified carcinomas. Given the inclusion of both primary and metastatic specimens, observed differences across subtypes should be interpreted with caution, as the site of sampling and prior treatment exposure were not available for adjustment.

### 3.1. Genomic Alteration Burden Across CACs Subtypes

Comprehensive genomic profiling of 276 CACs cases revealed substantial differences in the number and type of genomic alterations (GA) across tumor subtypes. Overall, the mean number of genomic alterations per tumor was significantly higher in sebaceous gland-derived tumors (SEB) compared to sweat gland-derived tumors (SWT), with SEB tumors harboring a mean of 7.9 alterations per tumor versus 4.9 alterations per tumor in the SWT cohort (*p* = 0.004). This difference underscores a notably higher mutational burden in SEB tumors relative to other adnexal tumor types. In contrast, digital papillary adenocarcinomas (DPA) demonstrated the lowest mean number of genomic alterations per tumor, with only 2.1 alterations per tumor compared to 5.0 alterations per tumor in non-DPA tumors (*p* = 0.03), reflecting a more genomically stable profile in this subgroup. Notably, analysis of genomic ancestry did not reveal significant differences between SEB and SWT tumors, suggesting that the observed differences in mutational burden were not confounded by patient ancestry.

### 3.2. Recurrent and Subtype-Specific Genomic Alterations

Detailed examination of key oncogenic events revealed that sebaceous tumors consistently harbored higher frequencies of critical tumor suppressor gene alterations. Specifically, RB1 alterations were detected in 38.2% of SEB tumors, compared to only 8.1% of SWT tumors (*p* < 0.0001). Similarly, TP53 mutations were identified in 76.4% of SEB tumors, substantially higher than the 43.4% observed in SWT tumors (*p* = 0.0002). The predominance of RB1 and TP53 alterations in SEB tumors suggests a potential neuroendocrine differentiation pathway and highlights the distinct molecular signature of sebaceous-derived CACs.

Among mucinous carcinomas (MC), the frequency of PTCH1 genomic alterations was significantly elevated compared to non-MC tumors, with 36.4% of MC tumors harboring PTCH1 alterations versus only 1.8% in other subtypes (*p* = 0.044). This finding indicates a potential subtype-specific pathway driving tumorigenesis in mucinous carcinomas.

### 3.3. Microsatellite Instability and Mismatch Repair Signatures

Analysis of microsatellite instability (MSI) status revealed that MSI-High (MSI-H) status was predominantly observed in SEB tumors, occurring in 15.7% of cases, whereas only 1.2% of SWT tumors exhibited MSI-H status (*p* = 0.005). In addition, the presence of mismatch repair (MMR) mutational signatures was significantly enriched in SEB tumors, with 32.0% of SEB tumors demonstrating MMR-related genomic signatures compared to only 2.1% of SWT tumors (*p* = 0.005). These findings collectively suggest that sebaceous tumors may possess a hypermutator phenotype, characterized by deficient DNA repair mechanisms, which further supports the presence of a hypermutator phenotype within this subgroup.

### 3.4. Genomic Loss of Heterozygosity

Assessment of genomic loss of heterozygosity (gLOH) further supported the distinct genomic landscape of SEB tumors. Tumors with gLOH greater than 16% were observed more frequently in the SEB cohort, with 19.6% of SEB tumors exhibiting elevated gLOH compared to 7.2% of SWT tumors (*p* = 0.081). While this difference did not reach conventional statistical significance, the trend suggests a greater degree of chromosomal instability in SEB tumors, which may contribute to their higher overall burden of genomic alterations.

### 3.5. Tumor Mutational Burden Across Subtypes

Tumor mutational burden (TMB) analysis demonstrated that most CACs subtypes harbored a moderately elevated mutational load. HRF tumors exhibited a mean TMB of 10.4 mutations per megabase (mut/Mb), while MC tumors showed the highest mean TMB at 38.8 mut/Mb. Conversely, APO tumors demonstrated a notably low mean TMB of 2.7 mut/Mb (*p* = 0.001), and DPA tumors exhibited the lowest TMB at 1.4 mut/Mb (*p* = 0.003). These findings highlight considerable variation in mutational load across subtypes, with sebaceous and mucinous tumors demonstrating the highest TMB and APO and DPA tumors demonstrating the lowest.

### 3.6. PD-L1 Expression

Immunohistochemical analysis of PD-L1 expression revealed generally low levels across all CACs subtypes. Tumor proportion scores (TPS) were 37.0% in SWT tumors versus 33.3% in SEB tumors, with no statistically significant difference observed (NS). This suggests that while PD-L1 expression is present in a subset of tumors, it is overall low and may not serve as a strong standalone biomarker for immunotherapy selection in CACs.

### 3.7. Summary of Key Findings

Taken together, these results demonstrate that sebaceous gland-derived CACs consistently exhibit a higher number of genomic alterations, a higher prevalence of key tumor suppressor gene mutations (TP53, RB1), elevated MSI-H status, enriched MMR signatures, and a trend toward increased gLOH compared to sweat gland-derived tumors. These findings establish SEB tumors as a genomically distinct subgroup within CACs, characterized by a higher burden of targetable or immunologically relevant alterations. In contrast, other subtypes such as APO and DPA demonstrate lower mutational burdens, fewer genomic alterations, and limited PD-L1 expression, highlighting the heterogeneity of molecular profiles within CACs.

Results are shown in Table 1.

## 4. Discussion

This study provides a large-scale descriptive analysis of the genomic landscape of clinically advanced CACs and should be interpreted as exploratory and hypothesis-generating. Cutaneous adnexal tumors (CATs) are a rare group of benign and malignant neoplasms arising from different adnexal structures of the skin, including hair follicles, sebaceous glands, and sweat glands [1,2,3,4,5,6]. Transformation to CACs is rare and presents significant diagnostic challenges due to the morphological and histological overlap with other skin tumors [1,3,15]. While a multidisciplinary diagnostic approach—incorporating clinical, histopathological, and immunohistochemical analyses—remains the standard for classification, relying on histopathology and immunohistochemistry alone is often insufficient for fully characterizing these tumors, particularly in advanced or metastatic settings [3,4]. In this study, our genomic analysis, along with two cases, highlights the importance of comprehensive genomic profiling (CGP) in distinguishing the molecular landscape of CACs and identifying clinically relevant biomarkers [5].

Comprehensive genomic profiling (CGP) of these tumors was used to define the molecular landscape of CACs. Understanding the genomic alterations in CACs is critical for advancing therapeutic strategies. While CGP has been used in multiple cancers to identify oncogenic mutations, recent studies in CACs have identified key oncogenic drivers and tumor suppressor alterations, including known mutations in tumor suppressor genes such as *TP53*, *RB1*, and *PTCH1* [2,6,16,17]. Notably, our findings align with prior studies indicating that sebaceous gland-derived CACs exhibit higher frequencies of *RB1* and *TP53* mutations, suggesting a potential link to neuroendocrine differentiation [6,16]. Additionally, we observed an elevated TMB in sebaceous CACs, further supporting the rationale for immunotherapy in these tumors. High TMB and MSI-high status have been associated with improved responses to immune checkpoint inhibitors in solid tumors, including colorectal cancer and NSCLC, making it an explorable option in sebaceous CACs [7].

Recent studies have significantly advanced our understanding of the molecular landscape of cutaneous adnexal carcinomas, revealing a remarkable degree of genomic heterogeneity across histologic subtypes. Porocarcinomas, one of the more extensively characterized subtypes, are defined by recurrent YAP1 gene fusions, most commonly YAP1-MAML2 and YAP1-NUTM1, which disrupt the Hippo signaling pathway [18,19]. The Hippo pathway plays a central role in regulating cellular proliferation, apoptosis, and organ size; its dysregulation through YAP1 fusions leads to unrestrained transcriptional activation of oncogenic programs. Importantly, these fusions have been confirmed not only in malignant porocarcinomas but also in benign poromas, suggesting that YAP1 rearrangements may represent an early oncogenic event that drives malignant transformation in a subset of tumors. In addition, NUTM1 rearrangements have been identified in other rare adnexal malignancies, including hidradenocarcinomas and malignant appendage tumors exhibiting cylindroma-like features [20]. This highlights the overlapping molecular mechanisms among different adnexal tumor lineages, indicating that certain oncogenic events, while rare, can contribute broadly to tumorigenesis across multiple subtypes.

Spiradenomas and spiradenocarcinomas, in contrast, display a distinct molecular profile. CYLD, a tumor suppressor that negatively regulates NF-κB signaling through deubiquitination of key pathway intermediates, is frequently inactivated in these tumors. Loss of CYLD function leads to aberrant NF-κB activation, promoting cell survival and proliferation. Interestingly, a subset of CYLD-wildtype tumors harbors recurrent ALPK1 hotspot mutations, providing an alternative mechanism for NF-κB pathway activation and suggesting convergent oncogenic pathways across spiradenocarcinomas. High-grade progression in these tumors is often accompanied by TP53 mutations, implicating additional genomic instability in driving aggressive clinical behavior [21]. Together, these observations underscore the complex, multi-layered nature of genomic alterations in adnexal carcinomas, with both lineage-specific drivers and secondary alterations shaping tumor phenotype and aggressiveness.

Our analysis of the Foundation Medicine Insights (FMI, Cambridge, MA, USA) database further demonstrates the rarity and heterogeneity of these events in advanced disease. NUTM1 rearrangements were detected in only four advanced cases—two porocarcinomas, one hidradenocarcinoma, and one malignant appendage tumor with cylindroma-like features—whereas MAML2, CYLD, and ALPK1 alterations were not observed [22]. This likely reflects a combination of biological factors, including the low prevalence of these alterations in metastatic or recurrent disease, as well as potential limitations of current profiling methods in detecting structural rearrangements or low-frequency variants. Such findings highlight the critical importance of comprehensive genomic profiling (CGP) in rare tumors, not only for capturing the full spectrum of actionable alterations but also for providing insights into tumor evolution, progression, and therapeutic vulnerability.

Extending these prior observations, our study provides additional insight into the molecular heterogeneity of metastatic CACs. Sebaceous gland-derived tumors, in particular, demonstrate a distinctive genomic profile characterized by frequent TP53 and RB1 alterations, reflecting disruption of key cell cycle checkpoints and DNA damage response pathways. These tumors also exhibit the highest incidence of microsatellite instability-high (MSI-H) status and mismatch repair (MMR) mutational signatures, consistent with a hypermutator phenotype. This genomic landscape suggests that sebaceous CACs represent a biologically distinct subset that warrants further investigation in prospective studies evaluating immune checkpoint inhibition. Although high TMB and MSI-H status have been associated with improved immunotherapy outcomes in other malignancies, this study does not include treatment response or survival data and therefore cannot establish predictive or prognostic utility in CACs.

By contrast, CACs originating from apocrine and eccrine sweat glands generally exhibit lower TMB and fewer genomic alterations, suggesting a more genomically stable phenotype and, potentially, a reduced intrinsic immunogenicity. Nevertheless, the occasional presence of actionable or targetable alterations in these subtypes emphasizes that CGP remains valuable even when the overall mutational burden is low. Identifying rare driver events or fusions may provide opportunities for targeted therapies or clinical trial enrollment, underscoring the importance of precision oncology approaches in all CACs subtypes.

The implications of these findings are multifold. First, they highlight the diagnostic utility of CGP in refining tumor classification, particularly in histologically ambiguous cases or tumors with mixed differentiation patterns. Second, they suggest a framework for personalized treatment strategies based on molecular features, including the potential prioritization of immunotherapy for MSI-H or high-TMB sebaceous tumors and exploration of targeted agents for rarer actionable alterations in other subtypes. Third, they emphasize the need for continued collection and analysis of molecularly annotated CACs cases, given the rarity of these tumors and the limited prospective clinical data available.

Finally, future research should aim to integrate comprehensive molecular profiling with longitudinal clinical outcomes to suggest predictive and prognostic biomarkers for CACs. Prospective clinical trials are critical to determine whether observed genomic alterations, including TP53, RB1, MSI-H status, and rare fusions, can guide therapeutic decision-making and improve patient outcomes. Moreover, deeper investigation into mechanisms of tumor progression, resistance to therapy, and immune evasion will be essential to fully translate molecular insights into clinically actionable strategies. Only through such integrative approaches can we hope to develop evidence-based, biomarker-driven guidelines for the diagnosis and management of these rare but clinically challenging tumors, ultimately advancing the precision oncology paradigm for patients with CACs.

**Figure 1:**
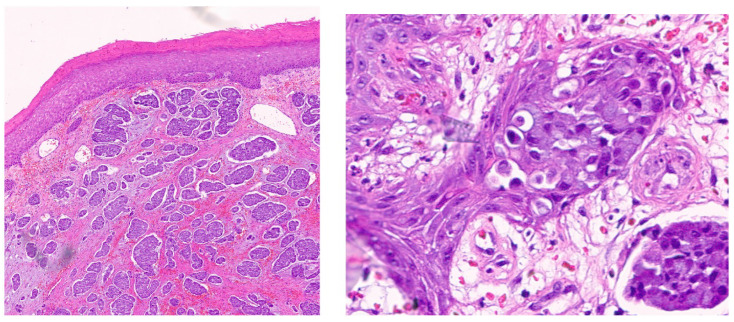
Primary sweat duct adenocarcinoma of the vulva (**left** histology image) with extra-mammary Paget’s Disease (**right** histology image). Hematoxylin and Eosin (H&E) staining at 200× (**left**) and 400× (**right**).

**Figure 2:**
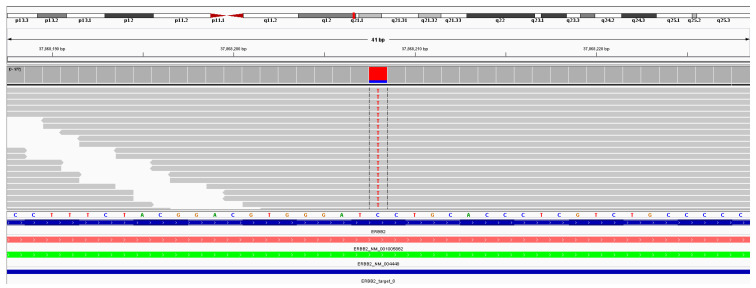
Comprehensive genomic profiling showing ERBB2 mutations.

To further illustrate the clinical implications of CGP in CACs, we describe two representative cases that highlight its potential to guide targeted treatment:

The first case involves a 73-year-old female patient with a primary sweat duct adenocarcinoma of the vulva (Figure 1), complicated by extra-mammary Paget’s disease, which progressed to stage IV disease. Genomic profiling revealed that her tumor was microsatellite stable (MSS) with a low tumor mutational burden (TMB) of 5 mutations/Mb. The tumor carried two *ERBB2* mutations: a juxtamembrane mutation (R678Q) and an extracellular domain mutation (S310F), Figure 2. Sweat gland adenocarcinomas with extracellular domain *ERBB2* mutations have been reported to respond to anti-HER2 targeted therapies, and this was supported by a sustained 6-month response observed on PET imaging [23].

**Figure 3:**
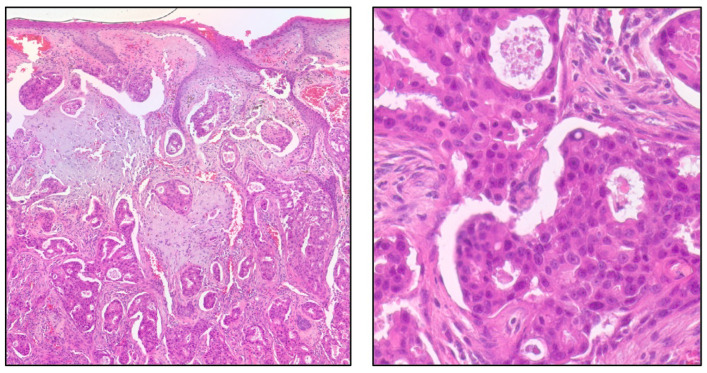
Apocrine sweat gland adenocarcinoma. Hematoxylin and Eosin (H&E) staining at 200× (**left**) and 400× (**right**).

**Figure 4:**
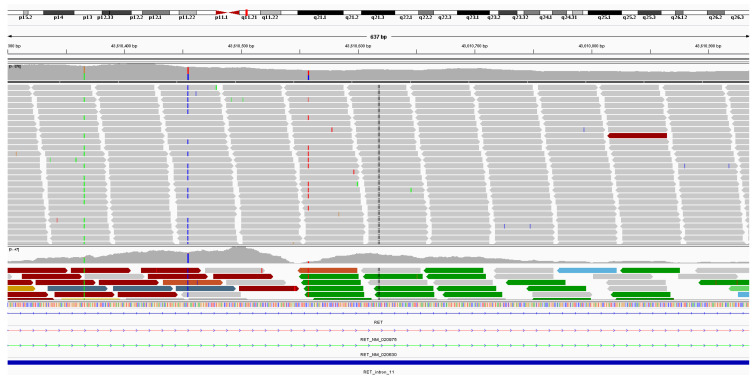
Comprehensive genomic profiling showing RET(NM_020630)-NCOA4(NM_005437) fusion (R11; N8).

The second case is a 65-year-old male patient with apocrine sweat gland adenocarcinoma (Figure 3) presenting as a growing tumor in the buccal area, with invasion of the underlying parotid salivary gland and involvement of several regional lymph nodes. Comprehensive genomic profiling demonstrated that the tumor was MSS with an undetectable TMB (0 mutations/Mb). Additional findings included loss of *CDKN2A/B* and *MTAP*, along with the presence of an *NCOA4-RET* fusion (Figure 4). Given the approval of selpercatinib for RET fusion-positive solid tumors, this finding identifies a potentially targetable alteration, although clinical efficacy in this disease context requires further validation [24].

These cases are presented to demonstrate potential clinical applications of CGP rather than to establish therapeutic efficacy. The identification of *ERBB2* mutations in one case and an *NCOA4-RET* fusion in another highlights the benefits of CGP in guiding targeted therapy, even in rare tumor types where standard treatment options are limited. The presence of mutations such as *ERBB2* and *RET* supports the application of approved targeted agents, offering a pathway toward more personalized treatment.

While PD-L1 expression was generally low across all tumor types and did not significantly differ between subgroups, the high TMB and MSI-high status in sebaceous tumors remain promising biomarkers for potential clinical trials [6,16]. Notably, ongoing trials in lung cancer and other solid tumors, including metastatic ones, are already investigating the efficacy of immunotherapeutic approaches in tumors with high TMB, highlighting the relevance of these biomarkers across cancer types. Moreover, the observed high prevalence of genomic alterations in sebaceous CACs compared with sweat gland tumors shows the importance of stratifying patients by histological subtype when considering therapeutic strategies. Future prospective, clinically annotated trials will be essential to determine whether the genomic alterations described here possess prognostic or predictive value.

An important consideration in interpreting these findings is the biological heterogeneity of the cohort. The inclusion of both primary and metastatic specimens, as well as multiple histologic subtypes, introduces potential confounding variables that could influence observed differences in genomic alterations, TMB, and MSI status. Without paired primary-metastatic comparisons or treatment history data, it is not possible to determine whether certain alterations reflect intrinsic subtype biology or evolutionary changes associated with tumor progression.

## 5. Limitations

Limitations of our study include its lack of direct clinical outcome data following targeted therapy or immunotherapy, which limits the ability to draw definitive conclusions regarding therapeutic efficacy. Additionally, the retrospective nature of the dataset and inclusion of both primary and metastatic tumor samples introduce heterogeneity that may influence the genomic findings. Furthermore, the Foundation Medicine Insights (FMI, Cambridge, MA, USA) database is restricted to clinically advanced (predominantly metastatic) tumors, which may underrepresent alterations more common in localized or indolent disease. Because primary and metastatic tumors were analyzed collectively without matched comparisons, subtype-specific differences may partially reflect stage-related genomic evolution. Nonetheless, our findings provide a foundational step toward a precision medicine approach for CACs, emphasizing the need for prospective clinical trials to validate the utility of CGP-driven treatment strategies.

## 6. Conclusions

In conclusion, our comprehensive study demonstrates that CACs, particularly those arising from sebaceous gland differentiation, exhibit a distinct spectrum of genomic alterations that may have meaningful clinical and therapeutic implications. Through extensive comprehensive genomic profiling (CGP), we observed that sebaceous-derived tumors display higher frequencies of specific driver alterations, a greater burden of somatic mutations, and a propensity for molecular signatures associated with mismatch repair deficiency and microsatellite instability. These findings underscore the biological heterogeneity of CACs, highlight the importance of molecular characterization in understanding tumor behavior, and may inform future biomarker-driven studies and provide a framework for future clinical investigation.

The integration of CGP into routine diagnostic workflows has the potential to significantly refine the histopathologic classification of CACs. Traditional morphologic assessment alone can sometimes be insufficient to fully capture the diverse differentiation patterns of these tumors, particularly in cases that are histologically ambiguous or unclassified. Incorporating genomic profiling not only provides an additional layer of diagnostic precision but also facilitates the identification of molecularly defined subgroups that may benefit from targeted therapeutic interventions. For example, tumors demonstrating high TMB or MSI-H status have been associated with improved responses to immune checkpoint inhibitors in other tumor types; however, the predictive value of these biomarkers in CACs remains to be determined. Recognizing such molecular features in CACs could therefore expand treatment options in a clinical setting where standardized therapies are currently lacking.

Furthermore, the cataloging of recurrent genomic alterations presented in this study provides an invaluable reference for clinicians and researchers alike. Sebaceous gland-derived CACs, in particular, demonstrated a notably increased frequency of key genomic alterations compared with tumors arising from other adnexal lineages. By compiling these alterations systematically, our work offers a foundation for the identification of potential therapeutic targets and biomarker-driven clinical trial design. Such a repository is essential given the rarity of CACs, which has historically limited the feasibility of large, prospective studies and contributed to a paucity of evidence-based treatment guidelines.

Moving forward, future investigations should aim to integrate comprehensive molecular profiling with longitudinal clinical outcomes to develop predictive and prognostic frameworks specific to CACs. Correlating specific genomic alterations with treatment responses, survival outcomes, and patterns of disease progression will be critical in translating molecular insights into actionable clinical strategies. Moreover, prospective clinical trials are urgently needed to evaluate the predictive value of CAC-based biomarkers, particularly in the context of immune checkpoint inhibitor therapy. Only through such rigorous clinical investigation can we begin to establish evidence-based guidelines that optimize patient care, improve outcomes, and inform the rational design of novel therapeutic approaches for these rare but clinically challenging malignancies. Importantly, the absence of clinical outcome data limits conclusions regarding prognostic or predictive significance, and prospective clinically annotated studies are required before translating these findings into treatment recommendations.

In summary, our study not only confirms the molecular complexity of CACs but also emphasizes the critical role of CGP in both refining diagnosis and informing therapeutic decision-making. The observed enrichment of genomic alterations in sebaceous gland-derived tumors, alongside high TMB and MSI-H status in selected cases, provides a rationale for considering targeted and immunotherapeutic approaches in this patient population. As the field continues to evolve, integrating molecular data with clinical insights will be essential to unlock the full potential of precision oncology for CACs, ultimately enabling the development of tailored, biomarker-driven treatment strategies for patients with these rare tumors.

## Figures and Tables

**Table 1:** Comparative Genomic and Clinical Characteristics of Cutaneous Adnexal Carcinoma Subtypes.

	SWT vs. SEB			SWT APO vs. SWT ECC			
	SWT *n* = 173	SEB *n* = 55	*p*-Value	APO *n* = 23	ECC *n* = 150	*p*-Value	HRF *n* = 14
Gender (% male)/Mean Age (yrs)	63.6%/63.7 y	67.3%/67.4 y	0.896	78.3%/72.1 y	61.3%/62.4 y	0.433	28.6%/52.4 y
GA/Tumor	4.9	7.9	0.005	3.8	5.1	0.306	4.3
*RB1*	8.1%	38.2%	<0.0001	0.0%	9.3%	0.551	14.3%
*TP53*	43.4%	76.4%	<0.0001	17.4%	47.3%	0.068	35.7%
MSI-high	1.2%	15.7%	0.005	0.0%	1.4%	1.000	0.0%
Mean TMB	17.0	22.8	0.577	2.7	19.1	0.001	10.4
TMB ≥ 10 mut/Mb	24.3%	40.0%	0.113	0.0%	28.0%	0.011	28.6%
MMR	2.1%	32.0%	0.005	0.0%	2.2%	1.000	0.0%

## Data Availability

Data is available upon reasonable request.

## Abbreviation

APO	Apocrine
CAT	Cutaneous Adnexal Tumor
CGP	Comprehensive Genomic Profiling
DPA	Digital Papillary Adenocarcinoma
ECC	Eccrine
ERBB2	Erb-B2 Receptor Tyrosine Kinase 2
GA	Genomic Alteration
gLOH	Genomic Loss of Heterozygosity
HRF	Hair Follicle
ICI	Immune Checkpoint Inhibitor
IHC	Immunohistochemistry
MC	Mucinous Carcinoma
CAC	Cutaneous Adnexal Carcinoma
MMR	Mismatch Repair
MSI	Microsatellite Instability
MSI-H	Microsatellite Instability-High
mut/Mb	Mutations per Megabase
NCOA4-RET	Nuclear Receptor Coactivator 4—RET Fusion
PD-L1	Programmed Death-Ligand 1
PET	Positron Emission Tomography
POR	Porocarcinoma
PTCH1	Patched 1 (gene)
RB1	Retinoblastoma 1 (gene)
SEB	Sebaceous
SPR	Spiradenocarcinoma
SRNG	Syringoadenocarcinoma
SWT	Sweat
TMB	Tumor Mutational Burden
UNC	Unclassified Carcinoma
UNK	Unclassified
